# Frontal Control Process in Intentional Forgetting: Electrophysiological Evidence

**DOI:** 10.3389/fnins.2017.00757

**Published:** 2018-01-11

**Authors:** Heming Gao, Mingming Qi, Qi Zhang

**Affiliations:** School of Psychology, Liaoning Normal University, Dalian, China

**Keywords:** directed forgetting, maintenance rehearsal, cognitive control, P2, N2

## Abstract

In this study, we aimed to seek for the neural evidence of the inhibition control process in directed forgetting (DF). We adopted a modified item-method DF paradigm, in which four kinds of cues were involved. In some trials, the words were followed by only a forgetting (F) cue. In the other trials, after a word was presented, a maintenance (M) cue was presented, followed by an explicit remembering (M-R) cue or an forgetting (M-F) cue. Data from 19 healthy adult participants showed that, (1) compared with the remembering cue (i.e., M-R cue), forgetting cues (i.e., M-F cue and F cue) evoked enhanced frontal N2 and reduced parietal P3 and late positive complex (LPC) components, indicating that the forgetting cues might trigger a more intensive cognitive control process and that fewer amounts of cognitive resources were recruited for the further rehearsal process. (2) Both the M cue and the F cue evoked enhanced N2 and decreased P3 and LPC components than the M-R or M-F cue. These results might indicate that compared with the M-R and M-F cues, both the M and F cues evoked a more intensive cognitive control process and decreased attentional resource allocation process. (3) The F cue evoked a decreased P2 component and an enhanced N2 component relative to the other cues (i.e., M-R, M-F, M), indicating that the F cue received fewer amounts of attentional resources and evoked a more intensive cognitive control process. Taken together, forgetting cues were associated with enhanced N2 activity relative to the maintenance rehearsal process or the remembering process, suggesting an enhanced cognitive control process under DF. This cognitive control process might reflect the role of inhibition in DF as attempting to suppress the ongoing encoding.

## Introduction

Intentionally ignoring or forgetting out-of-date information is essential for memory function (Anderson et al., 2004; Anderson and Hanslmayr, 2014). The processing of task-relevant information may be disrupted by irrelevant information. Intentional forgetting might be helpful in reducing this interference (Nowicka et al., 2011; Benoit and Anderson, 2012). Intentional forgetting is usually investigated by adopting an item-method directed forgetting (DF) paradigm. During the study phase, remembering or forgetting cues are provided randomly following each item. To-be-remembered (TBR) items are followed by remembering cues, and to-be-forgotten (TBF) items are followed by forgetting cues. Generally, TBR items show superior memory performance over TBF items (Bjork and Woodward, 1973; Basden et al., 1993). This effect is called the DF effect.

According to the selective rehearsal account, the DF effect is due to the selective rehearsal of TBR words (Basden et al., 1993; Sheard and MacLeod, 2005). If a remembering instruction is received, participants engage in an elaborate rehearsal. Successful intentional forgetting occurs owing to the passive decay of an unrehearsed memory trace (Bjork and Woodward, 1973; Basden et al., 1993; MacLeod, 1999). The attentional inhibition account argues that an active inhibition process is triggered by forgetting cues (Geiselman and Bagheri, 1985; Zacks et al., 1996). This inhibitory process might serve to cease the rehearsal process of TBF items or suppress the memory representation (van Hooff and Ford, 2011).

With the merit of high temporal resolution, the event-related potential (ERP) technique has been employed to explore the neural activity underlying DF (Paz-Caballero et al., 2004; van Hooff and Ford, 2011; Gao et al., 2016a). The P2 component has been associated with attentional allocation process, with enhanced attention resulting in increased P2 amplitudes (Thorpe et al., 1996; Bergström et al., 2007; Qi et al., 2016). Some DF studies found that a more positive frontal P2 component was evoked for remembering vs. forgetting cues (Cheng et al., 2012; Gao et al., 2016a), indicating that forgetting cues received fewer amounts of attentional resources. Some studies found that compared with remembering cues, forgetting cues evoked more positive ERPs over the frontal scalp but evoked less positive ERPs over the parietal scalp (Paz-Caballero et al., 2004; van Hooff and Ford, 2011). Recently, some studies found that forgetting cues evoked a more negative N2 over the frontal scalp but decreased P3 and late positive complex (LPC)components over the parietal scalp compared with remembering cues (Yang et al., 2012; Patrick et al., 2015; Gao et al., 2016a). These studies suggested that the enhanced frontal ERP activity associated with forgetting cues might reflect the attentional inhibition process.

In the typical item-method DF paradigm, participants are aware that each item has an equal possibility of being followed by a remembering or a forgetting cue. They do not engage in an elaborate rehearsal when the items are presented. These items are kept in working memory by rote rehearsal or maintenance rehearsal before cues are presented (Woodward et al., 1973; Greene, 1987). Therefore, different cognitive strategies might be adopted in response to different cues. Specifically, remembering cues trigger the elaborate rehearsal process for TBR items, while this process was absent for forgetting trials, in which TBF items are passively decayed or inhibited (Wylie et al., 2008). The ERP cue effect (remembering vs. forgetting cue) might reflect that participants adopted different cognitive strategies. There is no firm evidence showing that forgetting cues trigger the inhibition control process.

Previous studies have demonstrated that cognitive control over overt behavior is always associated with the activity of the frontal/prefrontal cortex. For example, this frontal activity was always observed in motor response inhibition tasks (e.g., Bokura et al., 2001; Aron et al., 2014) and switching tasks (e.g., Dove et al., 2000; Philipp et al., 2013). Goal-directed cognition generally requires cognitive control, and Anderson et al. (2004) suggested that an explicit forgetting instruction might place demands on controlled attention. Conway and Fthenaki (2003) found that the DF effect was diminished in patients with frontal lobe damage. Therefore, it seems that DF may involve the attentional/cognitive control process. Wylie et al. (2008) found that the neural activity associated with intentional forgetting differed from that associated with unintentional forgetting and intentional remembering, and they speculated that the maintenance rehearsal process is associated with the activity of the inferior frontal regions, and that the activity in the parahippocampal area may reflect the attempt to relate the maintained items to the remembering/forgetting instructions. However, no studies have investigated the neural activity associated with the maintenance rehearsal process in DF.

The present study focused on the neural activity of the maintenance rehearsal process. In the item-method DF procedure, the maintenance rehearsal process was interrupted by remembering and forgetting cues. According to the selective rehearsal account, maintenance rehearsal was passively ceased without any further cognitive processes acting upon the TBF information. However, the attentional inhibition account suggested that processing resources were actively withdrawn from the memory representation of TBF items, and attention was inhibited from returning to the memory representations of TBF items. Therefore, if DF is a passive process, decreased neural activity would be found for the forgetting process vs. the maintenance rehearsal process. On the contrary, if enhanced neural activity was found for the forgetting process vs. the maintenance rehearsal process, it might imply that the forgetting cues triggered an active inhibitory process.

**Figure 1:**
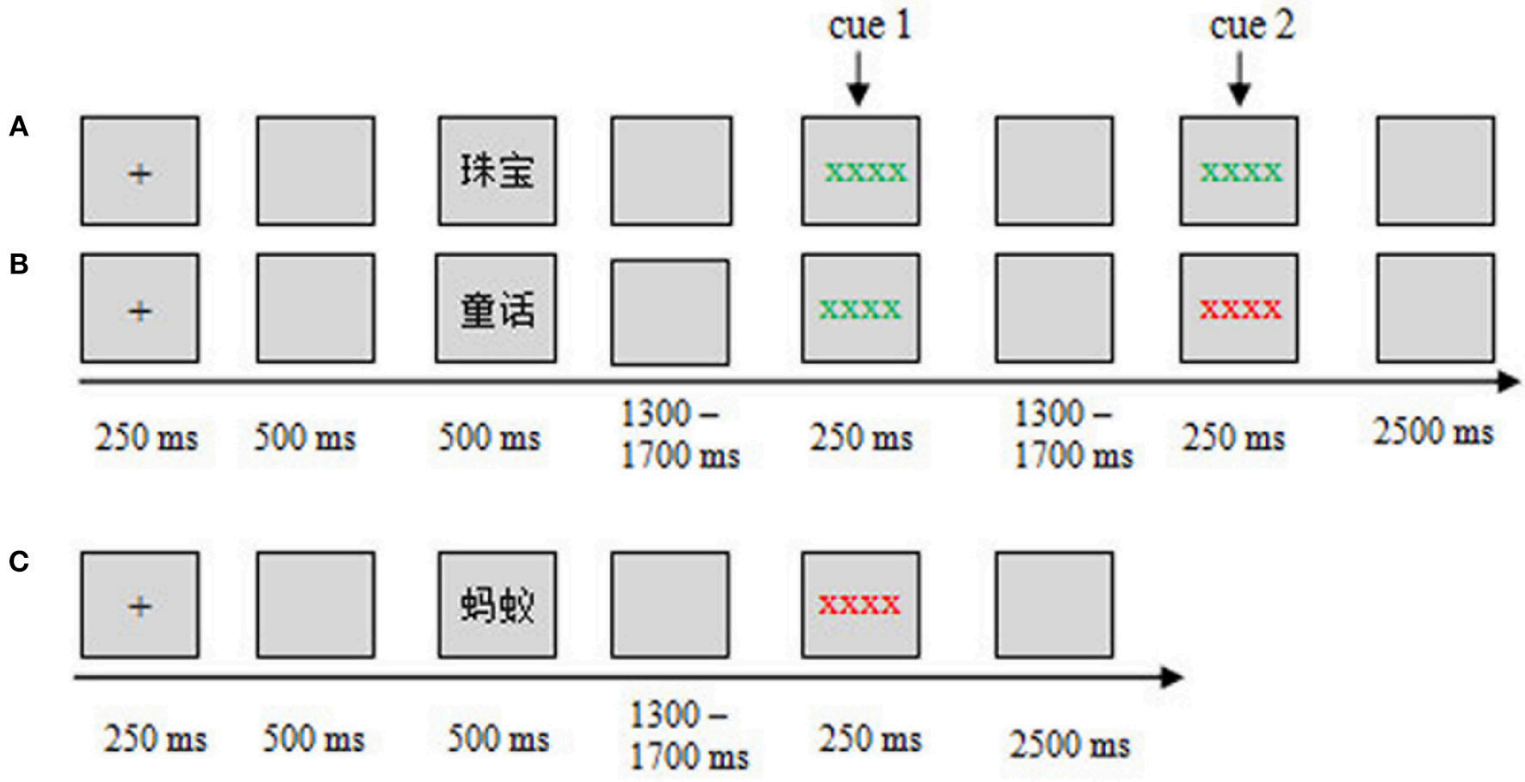
Experimental design and procedure. The sequence of events in the study phase for different conditions is shown: **(A)** M-R, **(B)** M-F, and **(C)** F. ERPs were time-locked to the cue onset.

In this study, we adopted a modified item-method DF paradigm, in which a maintenance (M) cue was presented before the remembering/forgetting cues (Figures 1A,B). Specifically, when participants saw the M cue, they could not know whether the word was TBR or TBF until the following cues (remembering M-R or forgetting M-F) appeared. Therefore, when the M cue was presented, the participants refreshed the words through maintenance rehearsal until the remembering/forgetting cue was presented. The M cues would trigger the maintenance rehearsal process. In the other trials, the word was followed by only a forgetting cue (F), and this word was categorized as a TBF item (Figure 1C). The F cues would trigger the DF process. By using this modified paradigm, we investigated the neural activity underlying maintenance rehearsal and DF (i.e., ERPs evoked by M and F cues).

Previous DF studies found that forgetting cues evoked more negative frontal N2 and less positive parietal P3 components compared with remembering cues (Patrick et al., 2015; Gao et al., 2016a,b). A similar ERP effect would be expected for the M-R vs. M-F cues in this study. As a subcomponent of the P3 component, P3a exhibits a fronto-central scalp distribution and has been associated with the reallocation of attentional resources. Therefore, an enhanced P3a would be found for the M-R cue relative to the M-F cue. In addition, it is necessary to note that the M cues always preceded the M-F/M-R cues, and encoding of item information might benefit from maintenance rehearsal, leading to greater memory trace strength for the M-R condition relative to the M-F condition. Therefore, we speculated that the ERP difference between the M and M-F cues might be modulated by the differential memory trace strength of the words. To eliminate this order effect, the third condition (F) was designed.

This study mainly focused on the ERP differences between the M and F cues. Previous DF studies found that forgetting cue evoked a decreased frontal P2 component compared with remembering cue (Cheng et al., 2012; Gao et al., 2016a). Similarly, in this study, we predicted that an enhanced P2 component would be observed for the M cues relative to the F cues. We hypothesized that if the memory representation of TBF words was passively decayed, the maintenance rehearsal process would be decreased after the F cues were presented. Therefore, the maintenance rehearsal process triggered by M cues would be more intensive than that triggered by F cues. The parietal P3 and LPC components are associated with the memory rehearsal process (Patrick et al., 2015; Gao et al., 2016b). Accordingly, enhanced P3 and LPC activities would be found for the M cues relative to the F cues. However, if the F cues triggered an active inhibition process to the memory representation of TBF words, the forgetting process might be more effortful than the maintenance rehearsal process. The frontal N2 component is known to be related to executive control (Espinet et al., 2012), inhibitory control of task-irrelevant information (Getzmann et al., 2015; Iannaccone et al., 2015), and the inhibitory process for TBF items (Bergström et al., 2007; Mecklinger et al., 2009; Patrick et al., 2015; Gao et al., 2016a,b). Levy and Anderson (2008) suggested that mechanisms engaged in attentional inhibition might be relate to cognitive control processes that are similar to those used to control overt behavior. Therefore, an enhanced frontal N2 component would be evoked for the F cues relative to the M cues in this study.

## Materials and methods

### Participants

Twenty undergraduate native Chinese college students took part in this experiment. Because of excessive artifacts in the electroencephalographic recording, one participant was excluded from the analysis (<50% trials were valid after artifact rejection). Therefore, data from 19 participants were included in the analyses (nine male and 10 female participants, mean age = 23.1 years, standard deviation = 1.85). All participants were right-handed and self-reported as healthy. All participants had normal or corrected-to-normal eyesight, and none were color blind. This study was approved by the Research Ethics Committee of Liaoning Normal University of China and was in accordance with the ethical guidelines of the Declaration of Helsinki. All participants have granted their written informed consent, and were paid on completion of the experiment.

### Design and materials

In the study phase, after the words were presented, the participants either received a maintenance cue followed by a remembering/forgetting cue (Figures 1A,B) or received only a forgetting cue (Figure 1C). Specifically, if a green cue (string of green Xs, cue 1) followed the word, the participants needed to see the following cue (cue 2) to judge this word as TBR or not. If a green cue followed (cue 2), it was a TBR word (Figure 1A); if a red cue followed (cue 2), it was a TBF word (Figure 1B). If a word was only followed by a red cue (cue 1), this word was a TBF word (Figure 1C), and no additional cues followed. Therefore, there were three conditions (three kinds of words): maintain-remember (M-R), maintain-forget (M-F), and forget (F). A within-subject design was used in this study. The three conditions were presented in a pseudo-randomized order, with the constraint that no more than three consecutive trials could be from the same condition. The assignment of color to the remembering cue or forgetting cues was counterbalanced across subjects.

The learning materials were Chinese double-character nouns, which were selected from the top 8000 words in “The Modern Chinese Frequency Dictionary” with a mean frequency of 7.144 per thousand. Words were assigned to six lists, each containing 60 words. The mean number of strokes and frequency of the words were matched across different lists. Half the lists were used as learning materials for the study phase, and the remaining lists served as new words (distractors) for the test phase. Additionally, two buffer words followed by remembering cues were presented at the beginning and end of the study phase, which were excluded from subsequent analyses. Except for the buffer words, 180 words (60 words per condition) were presented in the study phase. The test phase consisted of 180 old words (60 of each from the M-R, M-F, and F words) and 180 new words (distractors). The sequence of presentation for list sets was counterbalanced across participants. The words were printed in black (RGB: 0, 0, 0), whereas the cues (Xs) were printed in green (RGB: 0, 255, 0) or red (RGB: 255, 0, 0). All stimuli (words and cues, font size 28 pt) were presented on a silver-gray background (RGB: 192, 192, 192). The participants sat approximately 80 cm from a computer screen.

In the test phase, a recognition test was conducted for the participants. Specifically, if a word had presented in the study phase (i.e., both TBR and TBF words), the participants were asked to give an “old” response, or else, give a “new” response.

### Procedure

During the study phase, each trial began with a 250 ms fixation cross, followed by a 500 ms blank screen. Then, a word was presented for 500 ms. For the M-R or M-F condition, after a random blank screen of 1,300–1,700 ms, a maintenance cue (cue 1) appeared for 250 ms, followed by a 1,300–1,700 ms blank screen, then a remembering/forgetting cue (cue 2: M-R/M-F cue) appeared for 250 ms, followed by a 2,500 ms blank screen (Figures 1A,B); For the F condition, after a word was presented, a 1,300–1,700 ms blank screen was presented, then a forgetting cue appeared for 250 ms, followed by a 2,500 ms blank screen (Figure 1C).

In the test phase, each trial began with a 250 ms fixation cross, followed by a 500–800 ms blank screen, and then a word appeared for 1,500 ms. Next, a 1,000 ms blank screen was presented. The participants were asked to press “f” or “j” on the keyboard to make old/new responses to the word as quickly and accurately as possible. The key assignment for the old/new responses was counterbalanced among participants.

### Data analysis

#### Behavioral data

The old response rate was defined as the percentage of old responses in each condition (i.e., hit rates to M-R, M-F, and F words and false-alarm rates to foils). A repeated-measure analysis of variance (ANOVA) with the factor word type (M-R, M-F, F, new) was performed on the old response rate.

#### ERP recording and analysis

Brain electrophysiological activity was recorded from a 64-Channel EEG recording system (Brain Products, GmbH, Germany) with references on a central midline electrode. A vertical electrooculogram (EOG) was recorded with electrodes placed below the right eye. A horizontal EOG was recorded with electrodes placed on the right canthi. All interelectrode impedance was maintained below 5 kΩ. EEG and EOG were amplified using a 0.05–100 Hz bandpass filter and continuously sampled at 500 Hz for off-line analysis.

Raw EEG data were processed offline using BrainVision Analyzer version 2.0 (Brain Products, GmbH; Gilching, Germany). For the data analysis, ERPs time-locked to the cues onset (during the study phase) were re-referenced algebraically to the average of the left and right mastoids. After ocular correction (Gratton et al., 1983), EEGs were digitally filtered with a 30 Hz low-pass filter with a 24 bit analog-to-digital converter. ERPs for all cues during the study phase were then segmented into 1,000 ms epochs surrounding stimulus presentation and baseline-corrected with respect to 200 ms pre-stimulus. Trials contaminated with EOG artifacts (mean EOG voltage exceeding ±80 μV) or those with artifacts due to amplifier clipping, bursts of electromyographic (EMG) activity, or peak-to-peak deflection exceeding ±100 μV were excluded from averaging. EEGs recorded in all conditions were averaged separately for each participant. The mean numbers of trials retained after artifact rejection were as follows, M-R cue: mean = 42.2, *SD* = 6.7, range, 32–52; M-F cue, mean = 42.8, *SD* = 5.6, range, 31–52; M cue: mean = 95.2, *SD* = 12.8, range, 78–119; F cue: mean = 47.4, *SD* = 7.4, range, 35–58.

For the study phase, the P2 (120–180 ms), N2/P3 (200–400 ms), and LPC (500–800 ms) time windows were chosen for statistical analysis, which corresponded to the typical latency range of the P2 (Smid et al., 1999), N2 (Folstein and Van Petten, 2008), P3 (Polich, 2007), and LPC (Patrick et al., 2015; Gao et al., 2016b) components.

The grand-averaged ERPs (**Figure 3**) showed that the maximum ERP difference during the P2 epoch distributed over the fronto-central scalp. Preliminary inspection of the data indicated that there were no ERP differences between conditions in the P2 time window at parietal electrodes (P3, Pz, P4). Therefore, data from the anterior-central recording sites [three frontal electrodes (F3, Fz, F4) and three central electrodes (C3, Cz, C4)] were selected for statistical analysis and divided into three levels (left vs. middle vs. right) of the hemisphere. Repeated-measures ANOVAs with cue type (M, M-R, M-F, F), caudality (frontal, central), and hemisphere (left, middle, right) as within-subject factors were performed on the mean amplitudes during the 120–180 ms time period.

As shown in **Figure 3**, during 200–400 ms, the M and F cues evoked a frontal N2 component, whereas the M-R and M-F cues evoked a frontal N2/P3 complex. The N2/P3a was maximally recorded at frontal sites around 300 ms (Campanella et al., 2002). Therefore, the selected epoch corresponded to the typical latency range and scalp distribution of the N2/P3 complex. Additionally, M-R/M-F cues evoked a parietal P3 component during 200–400 ms. Therefore, data from the anterior-posterior recording sites [three frontal electrodes (F3, Fz, F4), three central electrodes (C3, Cz, C4), and three parietal electrodes (P3, Pz, P4)] were selected for statistical analysis and were factorized into three levels (left, middle, right) of the hemisphere. Repeated-measures ANOVAs with cue type (M, M-R, M-F, F), caudality (frontal, central, parietal), and hemisphere (left, middle, right) as within-subject factors were performed on the mean amplitudes during the 200–400 ms period.

During the LPC (500–800 ms) time window, data from the anterior-posterior recording sites (F3, Fz, F4, C3, Cz, C4, P3, Pz, P4) were factorized into three levels (left, middle, right) of the hemisphere. Repeated-measures ANOVAs with cue type (M, M-R, M-F, F), caudality (frontal, central, parietal), and hemisphere (left, middle, right) as within-subject factors were performed on the mean amplitudes during the 500–800 ms period.

For the ERP data, to avoid describing large amounts of statistical data concerning scalp distribution effects, only main effects or interactions that included the cue factors were reported. All effects with >1° of freedom were adjusted for sphericity violations by using the Greenhouse-Geisser correction. Main effects (or interactions) were subjected to Bonferroni-corrected pairwise comparisons (or simple effect test).

## Results

### Behavioral results

**Figure 2:**
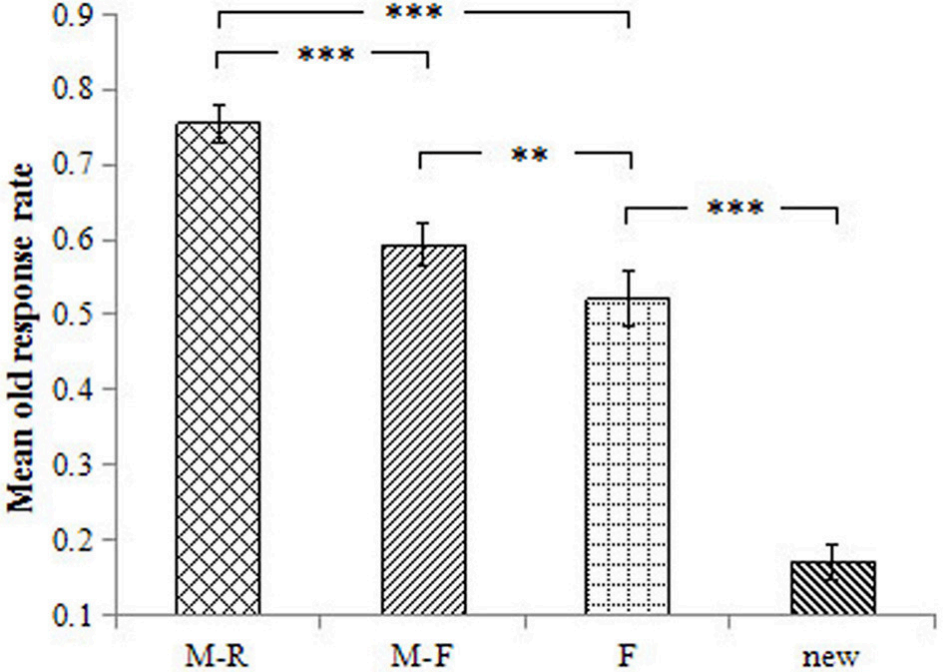
Mean old response rate for the different conditions. Error bars represent standard error of the mean. ^***^p < 0.001, ^**^p < 0.01.

The old response rate was 75.5% for the M-R words, 59.3% for the M-F words, 52.1% for the F words, and 17.0% for the new words. The ANOVA results revealed a main effect of word type on the old response rate, *F*_(3, 54)_ = 134.40, *p* < 0.001, $\eta_{p}^{2}$ = 0.882. Pairwise comparisons revealed that the old response rate was higher for M-R words than for all other words, *p*s < 0.001, *d*s ≥ 1.41. The old response rate was higher for M-F words relative to F words and new words, *p*s ≤ 0.001, *d*s ≥ 0.50; the old response rate was higher for F words than for the new words, *p* < 0.001, *d* = 2.66 (Figure 2).

### Electrophysiological results

During the P2 (120–180 ms) time window, the ANOVA results revealed a main effect of cue type, *F*_(3, 54)_ = 5.295, *p* = 0.003, $\eta_{p}^{2}$ = 0.226. Pairwise comparisons revealed that the F cue evoked smaller P2 amplitudes than the other types of cues (M-R, M-F, M), *p*s ≤ 0.027, *d*s ≥ 0.32. No interactions that included the cue factor were found, *p*s > 0.378.

During the 200–400 ms time window, the results revealed a main effect of cue type, *F*_(3, 54)_ = 51.90, *p* < 0.001, $\eta_{p}^{2}$ = 0.742. Both the Caudality × Cue type interaction [*F*_(6, 108)_ = 13.17, *p* < 0.001, $\eta_{p}^{2}$ = 0.422] and the Hemisphere × Cue type interaction [*F*_(6, 108)_ = 22.26, *p* < 0.001, $\eta_{p}^{2}$ = 0.553] were significant. Simple effect analyses revealed that the M-R cue evoked more positive ERPs relative to the other cues (M-F, M, F) over all scalps, *p*s ≤ 0.001, *d*s ≥ 0.39; the M-F cues evoked more positive ERPs relative to the M and F cues over the whole scalps, *p*s ≤ 0.004, *d*s ≥ 0.62; and the M cues evoked more positive ERPs relative to the F cues over all scalps, *p*s ≤ 0.019, *d*s ≥ 0.26.

During the LPC epoch (500–800 ms), the Cue type × Caudality × Hemisphere interaction was significant, *F*_(12, 216)_ = 1.91, *p* = 0.035, $\eta_{p}^{2}$ = 0.096. Simple effect analyses revealed that the M-R cues evoked more positive ERPs relative to M cues at central-parietal scalp electrodes (C3, Cz, C4, P3, Pz, P4), *p*s ≤ 0.028, *d*s ≥ 0.88; the M-F cues evoked more positive ERPs relative to M cues at central-parietal scalp electrodes (C3, Cz, C4, P3, Pz, P4), *p*s ≤ 0.048, *d*s ≥ 0.89; the M-F cues evoked more positive ERPs relative to F cues at C3 electrodes, *p* = 0.014, *d* = 0.98; and the M cues evoked less positive ERPs relative to the F cues at Pz and P4 electrodes, *p*s ≤ 0.026, *d*s ≥ 0.78.

### Correlational analyses

To better determine the functional means of observed ERP effects, Pearson correlation analysis was performed to investigate the association between the recognition accuracy (old response rate for M-R, M-F, and F words) and the amplitudes of the frontal N2 or the parietal P3 and LPC components. The amplitudes of the frontal N2 component were calculated by averaging the amplitudes from three frontal electrodes (i.e., F3, Fz, F4), as well as from three parietal electrodes (i.e., P3, Pz, P4) for the P3 and the LPC amplitudes. The results showed a positive correlation between the accuracy and the fronto-central N2 amplitudes, *r* = 0.544, *p* < 0.001. A positive correlation was found between the accuracy and the parietal P3 amplitudes, *r* = 0.409, *p* = 0.002. No correlation was found between the accuracy and the parietal LPC amplitudes, *r* = 0.09, *p* = 0.505.

## Discussion

The aim of the present study was to investigate whether forgetting is an active process that is more effortful than the maintenance rehearsal process. To trigger the maintenance rehearsal process, a maintenance cue was added into the item-method DF paradigm. The main findings are as follows: Consistent with previous DF studies, a typical DF effect was found. Memory performance benefited from the maintenance rehearsal process. ERP time-locked to the cues indicated that two continuous stages were involved in the forgetting process: the attentional withdrawal process, which was reflected by decreased frontal P2 activity, and the attentional inhibition process, which was reflected by increased frontal N2 activity. The enhanced frontal ERP activity associated with the forgetting process relative to the maintenance rehearsal process suggested that forgetting is an active process.

### Behavioral results

The memory performance was superior for the studied words (M-R, M-F, F) relative to the false-alarm rate for foils, indicating that all the studied words were reasonably encoded during the study phase. Consistent with previous DF studies (Bjork and Woodward, 1973; Basden et al., 1993), a typical DF effect was observed, with higher recognition performance for M-R words relative to M-F words, indicating an enhanced encoding process for M-R words during the study phase. This DF effect demonstrated that participants had successfully manipulated the words according to different (remembering, forgetting) instructions.

M-F words showed better memory performance relative to F words. In the M-F trials, the M cue did not indicate whether the words were TBR or not. Hence, the participants would continue to keep the words in working memory through maintenance rehearsal until the M-R/M-F (cue 2) was presented. In contrast, in the F trials, the forgetting cues were presented immediately after the words, and then the maintenance rehearsal process was reduced or terminated. Therefore, the maintenance rehearsal interval was greater for M-F trials than for F trials. Previous studies demonstrated that the encoding of item information benefited from maintenance rehearsal (Woodward et al., 1973; Hockley et al., 2016). Therefore, in this study, the better recognition performance for M-F words than that for F words might have benefited from the greater maintenance rehearsal interval. Most important, M cues might successfully trigger a maintenance rehearsal process.

### ERP results

The ERP technique is most commonly used in studies of memory (see the review by Rugg and Wilding, 2000), and the P3 and LPC components are always associated with memory manipulation (Polich, 2007; Gao et al., 2016b). The ERP technique is also particularly useful in investigating the neural activity associated with cognitive control. The N2 component, which is widely generated in the medial and lateral prefrontal cortex, is always associated with the cognitive control process (see the review by Folstein and Van Petten, 2008). With the advantage of time resolution, the ERP result could reveal the time course of the neural difference between different cues.

#### Cognitive control process triggered by forgetting cues: frontal N2 activity

**Figure 3:**
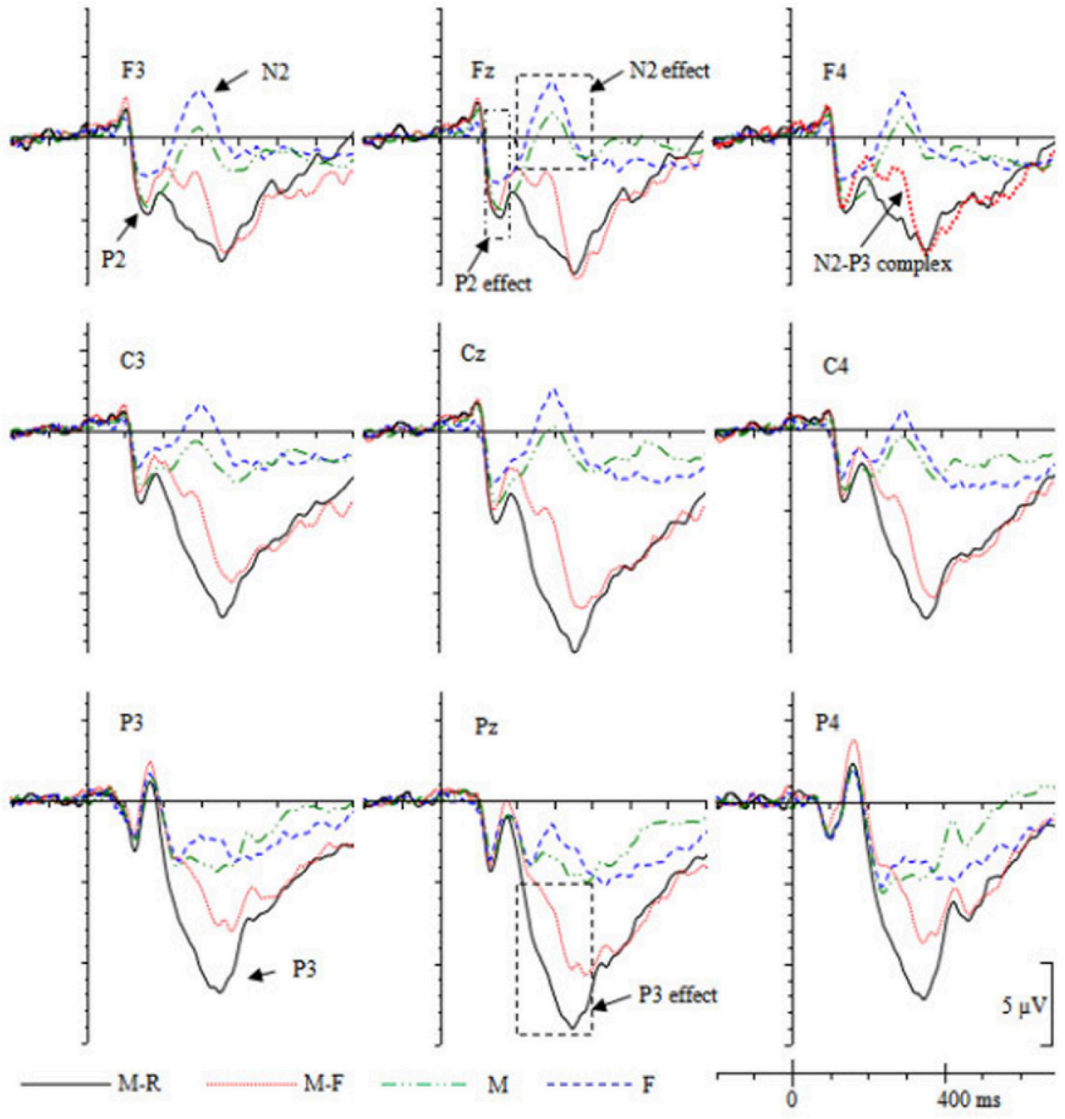
Grand averaged ERPs (*n* = 19) for different cues during the study phase.

Consistent with previous DF studies (Patrick et al., 2015; Gao et al., 2016a,b), the ERPs were more negative for the forgetting cue (i.e., M-F cue) relative to the remembering cue (i.e., M-R cue) during the 200–400 ms epoch. Specifically, the M-F cues evoked a frontal N2/P3 complex, which was absent for the M-R cues (i.e., a P3 component was evoked for M-R cues; see Figure 3). In addition, the F cue evoked a more negative N2 component relative to the M cue. Overall, forgetting cues were associated with enhanced N2 activity over the fronto-central scalp. This fronto-centrally distributed N2 component is usually observed on various measures of cognitive control, for example, the NoGo N2 (Bokura et al., 2001; Falkenstein, 2006) and the stop signal N2 (Kok et al., 2004). The N2 is often interpreted as reflecting cognitive control of attention, and N2 amplitudes were increased with attentional control improvement (Folstein and Van Petten, 2008; Espinet et al., 2012; Qi et al., 2017). Hourihan and Taylor (2006) argued that analogous to the process of preventing the implementation of an overt response, DF may involve a cognitive control process during overt encoding. The participants were encouraged to commit words to memory, and the forgetting instruction served to countermand this default covert action. In this study, the forgetting cues induced a similar fronto-central N2 activity as in motor stopping paradigms. This result might reflect a top-down cognitive control process in preventing TBF items from being further rehearsed.

The positive correlation between the fronto-central N2 amplitudes and the recognition accuracy revealed that enhanced fronto-central N2 activity was associated with decreased memory performance. It further confirmed the view that the N2 reflects the memory inhibition process. In this study, the forgetting cues (i.e., M-F and F cues) might trigger a more intensive cognitive control process relative to the remembering cues (i.e., M-R cues). Consequently, more amounts of cognitive resources would be allocated in further processing of M-R words relative to M-F and F words. This speculation was supported by the cue effect during the P3 time window.

#### Remembering cue-induced elaborate rehearsal process: parietal P3 activity

During the P3 epoch, the mean amplitudes were increased for the remembering cues (i.e., M-R cues) relative to the forgetting cues (i.e., M-F/F cues), indicating that TBR words received a more intensive rehearsal process. The observation of a significant positive correlation between recognition accuracy and the parietal P3 amplitudes indicates that the increased P3 amplitudes might be associated with an enhanced rehearsal process. This is in line with the findings of previous DF studies, which showed that enhanced central-parietal P3 activity was positively correlated with higher subsequent memory performance (Patrick et al., 2015; Gao et al., 2016a,b). The P3 component might be a neural mark of the rehearsal process.

During the 200–400 ms time window, the M cues evoked a frontal N2 component, whereas this component was absent for the M-R cues. Instead, a P3 component was evoked (Figure 3). In addition, the M-R cues evoked enhanced central-parietal P3 and LPC components relative to the M cues. These results might indicate that the M cues triggered an enhanced frontal control process, and more amounts of cognitive resources were recruited for the elaborate rehearsal of M-R words. When the M cues were presented, the participants continued to keep the words in working memory through maintenance rehearsal, and these words did not receive elaborate rehearsal until the M-R cues were presented.

#### Forgetting process vs. maintenance rehearsal process

Enhanced frontal N2 and decreased central-parietal P3 and LPC activities were also found for the M cue relative to the M-F cues, indicating that a more intensive cognitive control process might be triggered by the M cues (enhanced N2), and fewer amount of cognitive resources were recruited for further rehearsal (decreased P3 and LPC). Lee and Lee (2011) found that the memory performance was improved with increased forgetting cue duration (i.e., 1 vs. 5 s). The TBF items still received processing after the cues were presented. Consistent with this view, the enhanced P3 and LPC activities associated with the M-F cue might be due to this automatic rehearsal process, which was more intensive than the maintenance rehearsal process. Similarly, this ERP effect was also observed between the F cue and the M-F cue, indicating that M-F cues received enhanced rehearsal process.

However, because the M and F cues always preceded the M-R and M-F cues, the ERP cue effect might be modulated by this cue order. The more positive-going amplitude in the M-R/M-F condition might be due to the more negative slow waves in the baseline period (−200 to 0 ms) compared with the M condition. Maintenance of information in working memory has previously been related to negative slow waves (for a review, see Ruchkin et al., 2003; Drew et al., 2006). During the baseline time window, it is consequently possible that the ERPs are more negative for the M-R cues relative to the M cues. This potential difference in ERP amplitudes in the baseline period makes it difficult to compare the M-R and M conditions with each other, and could potentially explain the more positive-going ERPs in the M-R condition. Therefore, the ERP difference between M-F and M cues is not a good indicator to reflect the neural differences between the maintenance rehearsal and forgetting processes.

Because both the F cue and the M cue were the first cues after the words were presented, the ERP effect (F cue vs. M cue) was not influenced by the cue order effect. The F cues triggered a forgetting process and the M cues triggered a maintenance rehearsal process. Compared with the M cues, the F cues evoked a decreased fronto-central P2 component. This frontal P2 effect was also found for the F cues relative to the M-R or M-F cues. This fronto-central P2 effect reflects the enhanced selective attention process triggered by the task-relevant stimulus (Smid et al., 1999; Bergström et al., 2007; Mecklinger et al., 2009). Some DF studies also found that remembering cues evoked an enhanced frontal P2 component than forgetting cues (Cheng et al., 2012; Gao et al., 2016a). Consistent with these findings, our study suggests that increased attentional resources might be allocated to the M cues vs. the F cues.

The F cue evoked a more negative fronto-central N2 component relative to the M cues, suggesting that the F cues triggered an enhanced control process than the M cues. In addition, the ERPs were more positive for the M cues relative to the F cues over the parietal scalp during the 200–400 ms time window. As shown in Figure 3, at P3 and Pz electrodes, the F cues evoked an N2 component, whereas the M cues evoked a P3 component. This might indicate that the words continued to receive a rehearsal process when the M cue was presented. However, the rehearsal process was stopped or reduced when the F cue was presented. The enhanced frontal activity might provide evidence for the attentional inhibition account, which suggests that forgetting might involve an active attentional/cognitive control process.

Some researchers found that decreased P2 and enhanced N2 components were evoked for forgetting vs. remembering cues (Gao et al., 2016a). They demonstrated that DF involves two stages. During the first stage, the attentional withdrawal process stops the TBF items from being further processed; during the second stage, the memory representations of TBF items were inhibited. Similarly, in this study, decreased P2 and enhanced N2 amplitudes were evoked for the forgetting cue (i.e., F cue) relative to the M cue. This demonstrated that the forgetting cue-induced frontal activities (P2, N2) were not modulated by the remembering cue-induced elaborate rehearsal process. It further supported the view that forgetting is an active process that involves attentional withdrawal (P2 epoch) and cognitive control (N2 epoch).

The M cues evoked reduced LPC amplitudes relative to the F cues at parietal electrodes (i.e., Pz, P4), suggesting that the TBF words received enhanced, further processing after the F cues were presented. One possibility is that it might reflect enhanced working memory maintenance and/or rehearsal process for the F condition relative to the M condition. Lee (2012) suggested that TBF words were automatically processed to the extent that cognitive resources remained available. In addition, some studies have found that the forgetting cue in an item-method DF paradigm might prompt subjects to process the TBF items (Zwissler et al., 2015; Gao et al., 2016b). Therefore, the ERP cue effect during the LPC epoch might reflect that TBF words were automatically rehearsed, and more amounts of cognitive resources were recruited for the F cues relative to the M cues. However, no significant correlation was found between the recognition performance and LPC amplitudes. An alternative explanation for this LPC effect might be that, in order to remember as many TBR words as possible, the participants might cumulatively rehearse the TBR words from preceding trials when the forgetting cue was presented (Fawcett and Taylor, 2008, 2012). This LPC activity might be associated with the study phase retrieval or cumulative rehearsal process.

Zwissler et al. (2015) suggested that frontal brain activation associated with forgetting cues might result from either non-inhibitory processes, such as attention orienting, conflict monitoring, or unsuccessful inhibition attempts. The frontal activity (i.e., P2, N2 activity) found in this study might reflect the attention orienting and cognitive control processes. MacLeod (2007) defined cognitive inhibition as “the stopping or overriding of a mental process, in whole or in part, with or without intention.” In consideration of this definition, the present findings might reveal the role of inhibition in DF as attempting to suppress the ongoing encoding, although the TBF words were automatically processed.

There was a limitation to this paradigm. There were twice as many M cues as F cues in this study. In other words, for the participants, after the words were presented, the F cues showed a lower probability of occurrence relative to the M cues. Therefore, the magnitude of the cue effect (F cues vs. M cues) during the N2 epoch might be enhanced owing to this oddball effect, although the maximum oddball N2 difference distributed over the posterior rather than the anterior scalp (see the review by Folstein and Van Petten, 2008). Additionally, previous ERP studies employing the oddball paradigm demonstrated that rare stimuli evoked enhanced P3 and LPC activity relative to frequent stimuli (Campanella et al., 2002; Denecke et al., 2004). Therefore, the cue effect between F and M cues during the P3 and LPC time windows might also be affected by the oddball effect. Future studies could adjust the proportion of different trials to eliminate this oddball effect.

## Conclusions

This study aimed to compare the neural activity of maintenance rehearsal vs. DF. Compared with the M cue, the F cue evoked a decreased frontal P2 component and an enhanced frontal N2 component, indicating that DF is an active process that involves a frontal control process. In addition, DF might be more effortful relative to maintenance rehearsal. Furthermore, the cognitive control process might play an important role in intentional forgetting.

## Author contributions

HG and MQ: designed the experiment. HG: conducted the experiment and analyzed the data. HG and MQ: wrote the manuscript. All authors edited and revised manuscript and approved final version of manuscript.

### Conflict of interest statement

The authors declare that the research was conducted in the absence of any commercial or financial relationships that could be construed as a potential conflict of interest.
